# Effect of desmopressin on bleeding outcomes after native renal biopsy: a systematic review and meta-analysis

**DOI:** 10.1038/s41598-025-24092-7

**Published:** 2025-11-07

**Authors:** Ammar Yasser Ali, Rem Ehab Abdelkader, Rashad G. Mohamed, Mohammed. N. Abdelaziz, Radwa M. Abdelsattar, Ahmed R. A. Moustafa, Mohamed Rizk Elsayed, Bassant Barakat, Emad Samaan

**Affiliations:** 1 Mansoura Manchester Program for Medical Education, Faculty of Medicine, Mansoura University, Mansoura, Egypt; 2 Faculty of Medicine, Mansoura University Hospital, Mansoura, Egypt; 3 Associate Professor of Nephrology, Mansoura Faculty of Medicine, Mansoura, Egypt; 4 Mansoura Manchester Research Society Academic Lead, Mansoura University , Mansoura, Egypt

## Abstract

Percutaneous kidney biopsy is a key diagnostic tool in nephrology but carries a risk of post-procedure bleeding, with complications ranging from minor hematomas to life-threatening hemorrhages. Desmopressin (DDAVP), a synthetic vasopressin analogue, has been used as a prophylactic agent to reduce the risk of bleeding. This systematic review and meta-analysis aims to resolve uncertainties by evaluating efficacy and safety outcomes in diverse cohorts, informing evidence-based practice for desmopressin use in native renal biopsy care. A comprehensive literature search was conducted across PubMed, Web of Science, Scopus, Cochrane Library, and ClinicalTrials.gov, covering all records up to May 2025 for randomized controlled trials that compared Desmopressin with placebo before native kidney biopsy. The primary outcome was bleeding events following kidney biopsy, with secondary outcomes focusing on adverse effects associated with Desmopressin use. A total of five randomized controlled trials (RCTs), including 717 patients undergoing native renal biopsy, were included in our study. The study found that desmopressin was significantly effective than placebo in reducing total bleeding events in 157 participants (RR 0.51, 95% CI [0.31, 0.83], *P* = 0.008). However, there was substantial heterogeneity, with no significant improvement in hematoma formation or blood transfusion needs. The safety parameters, including flushing incidence, systolic and diastolic blood pressure, and hemoglobin, were not significantly different between the desmopressin and placebo groups. The point estimates for the endpoints were generally consistent with no clinically significant difference between treatments. Evidence available suggests that while desmopressin reduces the cumulative incidence of bleeding after native renal biopsy, its negligible effect on clinically significant complications does not support its routine use in this setting. It did not affect clinically important endpoints such as hematoma formation or need for transfusion, and no difference in safety between the two groups was noted. It is concluded that there is no existing evidence to support the routine premedication with desmopressin before native renal biopsy, but in certain high-risk cases, its use may be contemplated until further research is pending. Further, adequately powered randomized trials are required to establish a definitive therapeutic benefit.

**Supplementary Information:**

The online version contains supplementary material available at 10.1038/s41598-025-24092-7.

## Introduction

Percutaneous renal biopsy remains an important diagnostic tool in nephrology, providing a histopathological description of kidney disorders that dictates therapy and prognosis^1,2^. The procedure holds challenges, with concerns from post-biopsy bleeding being the most prevalent and clinically important adverse event. Bleeding complications can range from benign microscopic hematuria, which occurs in virtually all biopsies, to severe retroperitoneal hematomas requiring transfusion in 0.9–9% of cases and 0.4–0.6% of patients requiring an interventional angiography^1–3^. These bleeding concerns account for a large portion of healthcare costs, resulting in longer hospital stays and the necessity for invasive procedures. Although there are several causes of bleeding in post-renal biopsy, the kidney’s vascular architecture is most likely responsible for its distinct proclivity for bleeding^4,5^. This risk is exacerbated in individuals with chronic kidney disease (CKD), where uremic toxicity has been demonstrated to cause platelet dysfunction and lead to variations in adhesion, aggregation, and granule release. Uremic toxicity in CKD patients produces platelet dysfunction by increasing nitric oxide, cyclic GMP, and uremic toxins^5,6^. Moreover, anemia of chronic disease is common in CKD, which exacerbates hemostatic balance by inducing altered platelet margination due to decreased red blood cell number^5,6^.

Clinical risk stratification models have identified serum creatinine values > 2 mg/dL (OR 2.56, 95% CI 1.48–4.42) and hemoglobin in the 150 μmol/L range as indicators of what demonstrated a 60% relative risk reduction in bleeding complications with subcutaneous DDAVP (0.3 μg/kg). At the same time, a 2024 prospective study on intranasal DDAVP in CKD patients (eGFR ≤ 45 mL/min/1.73 m^2^) reported an increase in minor bleeding events (*p* = 0.02) but no difference in major complications^3,7^. Such discrepancies may be the result of differences in delivery routes, specifically intravenous vs. intranasal, and variations in patient selection, particularly the exclusion of dialysis-dependent patients in many studies^5,7^.

The purpose of this systematic review and meta-analysis is to clarify areas of uncertainty, specifically evaluating efficacy and safety outcomes in diverse cohorts, as well as to inform evidence-based practice guidelines for the use of desmopressin in the context of renal biopsy care and the populations most likely to benefit from prescriptive prophylactic DDAVP. The results will help to guide evidence-based, customized bleeding prophylaxis in nephrology practice.

## Methodology

### Protocol registration

By the Cochrane Handbook for Systematic Reviews of Interventions^8^, this systematic review and meta-analysis were carried out and preregistered in the International Prospective Register of Systematic Reviews (PROSPERO) with registration number (CRD420251055111), and followed the standards for the Preferred Reporting Items for Systematic Reviews and Meta-Analysis (PRISMA) statement^9^.

### Data sources & search strategy

A detailed literature review was conducted using pertinent keywords to identify publications published up to May 2025 in the following five databases: PubMed, Cochrane, Web of Science, Scopus, and ClinicalTrials.gov. Comprehensive search tactics are included in the Appendix to the Supporting Information. In addition to the core database searches, we checked the reference lists of all recognized publications and reviews. Furthermore, authors of relevant publications were approached for any additional published or unpublished work.

### Eligibility criteria

Studies were included if they met the following criteria: (1) randomized controlled trials (RCTs); (2) conducted in adult patients only undergoing native kidney biopsy; (3) comparing Desmopressin to placebo; and (4) reporting at least one relevant outcome. Studies were excluded if they (1) involved non-RCT research designs, (2) included pediatric populations, or (3) did not report outcomes of interest. To be clinically relevant, we included studies only specifying pharmacologically active (therapeutic) doses of desmopressin. Studies reporting on homeopathic, or ultra-low doses with no plausible hemostatic effect were deemed ineligible because of the potential risk of introducing clinical and methodological heterogeneity, which would compromise the interpretation of the meta-analytic results.

### Study selection and data extraction

Before adopting the Rayyan software^10^, search results were loaded into EndNote software^11^, which was used to identify and remove duplicates. To determine if any search results were suitable for the study, three authors independently reviewed the abstracts and titles of each result. Disagreements were settled through discussion. Articles meeting the inclusion criteria underwent complete text screening. Any pertinent studies overlooked in the first search were found by looking through the reference lists of the chosen research. Data from the final included studies, including publication year, target population, baseline characteristics, study details, and results, were manually extracted into an Excel spreadsheet. We defined primary bleeding outcomes based on standardized and clinically relevant criteria to enable comparability between the studies covered. Total bleeding events: It is a composite measure that encompasses all the described post-native kidney biopsy bleeding complications, both minor and significant. Minor bleeding is made up of clinically insignificant events such as small perinephric or subcapsular hematomas confirmed only by imaging and without clinical sequelae, or transient microscopic hematuria. Major bleeding: Clinically significant Bleeding, as characterized by one or more of the following: decrease in hemoglobin concentration ≥ 2 g/dL, transfusion of blood, hemodynamic disturbance secondary to bleeding, or appearance of large or expanding hematomas necessitating intervention (radiologic or surgical)^12–14^. This definition is in accordance with the standards applied in previous nephrology trials and adapted from baseline scales, such as those of the International Society on Thrombosis and Hemostasis (ISTH), and previous renal biopsy complication trials, where definitions varied across trials. We set outcomes reported as close as possible to these categories for pooled analysis.

### Risk of bias and certainty of evidence

The risk of bias in each RCT was evaluated using the Cochrane Collaboration’s tool for assessing the risk of bias in randomized trials (Rob2)^15^. Two authors independently assessed the risk of bias. Any disagreements were resolved through discussion.

### Assessed outcomes

Post-biopsy bleeding events were our main research outcomes. These included: (1) total bleeding events; (2) gross hematuria; (3) perinephric hematoma verified by radiology; (4) requirement for blood transfusion; and (5) need for interventional or radiological procedures. Any side effects of desmopressin administration, such as flushing, changes in hemoglobin levels as a marker for hemodilution, hypotension, or any other symptoms noted by the included trials, were considered secondary outcomes. Every outcome was assessed at the final endpoint of each study. The certainty of the evidence was evaluated using the GRADE system (Grading of Recommendations, Assessment, Development, and Evaluation). Based on this approach, the quality of the evidence was categorized into high, moderate, low, or very low certainty.

### Statistical analysis

RevMan software version 5.4.1^16^ was used to conduct the meta-analysis. Continuous outcomes were presented as mean differences (MD) with a 95% CI, whereas dichotomous outcomes were presented as risk ratios (RR). Due to the anticipated heterogeneity between studies, a random-effects model using the DerSimonian and Laird (DL) method was employed instead of a fixed-effects model to yield a more conservative estimate of the pooled effect. The I-square statistic was used to gauge the degree of heterogeneity, and the Chi-square test was used to verify its existence. The Cochrane Handbook (Chapter 9)^17^ defines substantial heterogeneity as an I-square value greater than 50% and considerable heterogeneity as a Chi-square test *p* value less than 0.1. A *p* value of less than 0.05 was considered statistically significant. Additionally, to address potential heterogeneity, sensitivity analyses were employed to evaluate the result’s robustness and to assess the contribution of each study by excluding studies one at a time. Because of the heterogeneity in desmopressin dosing (0.3–3 mcg/kg) and modes of administration (subcutaneous and intranasal) in the included studies, all quantitative syntheses were conducted using a random effects model, which allowed for differences in the exposures to the interventions between the studies and therefore provided more conservative pooled estimates. Clinical and methodological heterogeneity were preliminarily evaluated using I^2^ statistics and the Chi-square test. In instances of significant heterogeneity (I^2^ > 50%), we completed sensitivity analyses by removing studies from the meta-analyses that used different dosing strategies, administered different routes, or reported on patients who comprised different risk profiles (e.g., preserved vs. impaired renal function) to evaluate the robustness of the pooled effects. The limited number of eligible studies prevented us from properly assessing formal meta-regression or subgroup stratification based on dose or route.

## Results

### Literature search and study selection

**Fig. 1:**
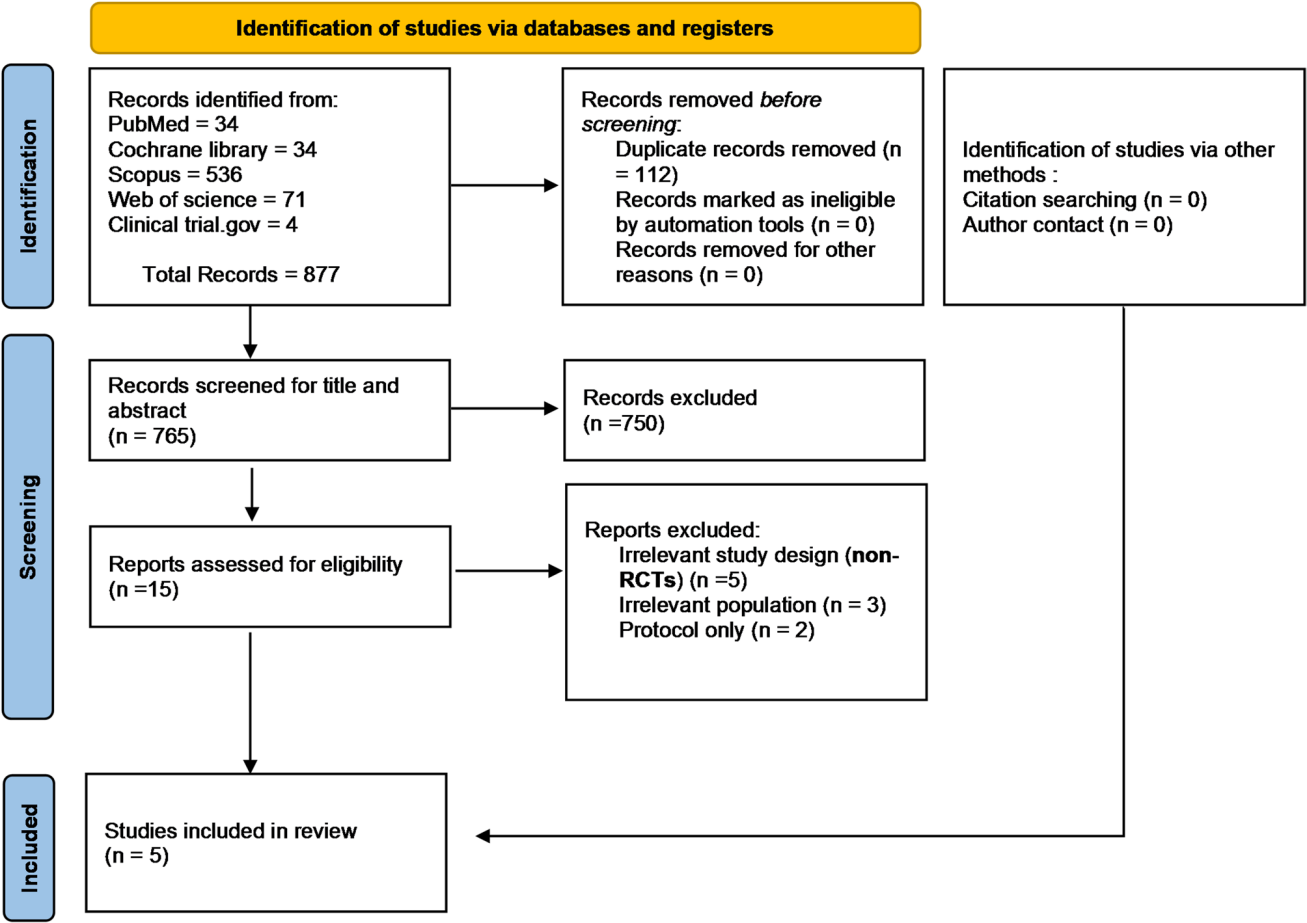
The Prisma flow chart illustrates the selection of studies for this review.

A systematic database search identified 877 potentially relevant records. After removing 112 duplicates using EndNote software^11^, 765 records underwent title and abstract screening, resulting in 15 articles. Following full-text review, 10 entries were removed for a variety of reasons. As a result, five studies (Chakrabarti^18^, Manno^19^, Prasad^20^, Sattari^21^, and Sethi^7^) were considered for both quantitative and qualitative analysis (Fig. 1).

### Characteristics of included studies

The included studies were summarized in order to provide an overview of their key characteristics, as outlined in Tables 1, 2. These tables provided essential data such as study design, sample size, population details (demographics and health status), intervention details and comparator details, and outcome measures. All studies were in randomized controlled designs. The studies involved a variety of participant populations, which varied in terms of age, sex, health status, and geography. The interventions and comparators were described in enough detail to consider comparisons and grouping of studies for synthesis. Outcome measures were mostly consistent across studies, aligned with relevant primary endpoints for the review question. The tables allowed for PICO (Population, Intervention, Comparator, and Outcome) comparisons and specifically highlight similarities and differences that may affect our synthesis and interpretation of the findings. The summary tables provide a framework for transparency and allow for assessment of the translatability of evidence to a clinical or specific research context (Table 3).

**Table 1:** Summary characteristics of the included studies.

Study ID	Study Identification			Inclusion criteria	Desmopressin group			Control		Follow-up duration	Primary outcomes	Secondary outcomes	Main conclusion
	Study design	Location	Start date–end date		n =	Dose/route	Timing of administration	n =	Type				
Chakrabarti (2025)	Single-center, double-blinded, randomized controlled trial (RCT)	India	January 2023–December 2023	Age > 18 years Controlled blood pressure eGFR ≥ 60 mL/min/1.73 m^2^ Undergoing a native kidney biopsy	74	3 mcg/kg/intranasal	1 h before the kidney biopsy	78	Saline	24 h	The occurrence of major or minor bleeding events following biopsy	The size of the perinephric hematoma at 6 h and 24 h after the biopsy	In patients with impaired renal function, desmopressin did not decrease the incidence of bleeding complications or post-biopsy hematoma formation
Prasad (2025)	Double-blind, randomized, placebo-controlled trial	India	February 2019–January 2023	Adults aged 18–65 years Undergoing a native kidney biopsy Any baseline eGFR is accepted	101	300 µg, intranasal	1 h before the procedure	102	Saline	24 h	Incidence of any post-biopsy bleeding event (major or minor)	Hematoma, hematuria, hemoglobin drop > 1 g/dL, hypotension, transfusion, and radiological intervention	Desmopressin significantly decreases post-biopsy bleeding and hematoma development, with tolerable side effects supporting its use as a preventive measure in renal biopsies
Sethi (2024)	Prospective randomized double‐blind pilot study	India	January 2021–September 2022	Adult patients Underwent native percutaneous kidney biopsy for any indication eGFR ≤ 45 mL/min/1.73 m^2^	40	150 μg intranasal	1 h before biopsy	40	Saline	8 h	Incidence of major post-biopsy bleeding (hemoglobin drop requiring transfusion, arteriovenous fistula, radiological intervention, or nephrectomy)	Incidence of minor bleeding (hematuria and hematoma on post-biopsy ultrasound)	Desmopressin elevated post-biopsy bleeding relative to placebo, demonstrating no preventive advantage, and further large-scale studies in dialysis patients are advised
Sattari (2022)	Double-blind randomized clinical trial	Iran	July 2017–August 2020	Age 18–80 years eGFR between 15 and 90 mL/min/1.73 m^2^	60	3 μg/kg intranasal	1 h before biopsy	60	Sodium chloride	24 h	Incidence of post-biopsy bleeding complications (major: AV fistula, acute renal obstruction, transfusion, embolization, sepsis, or death; minor: perirenal hematoma, gross hematuria)	Hematoma volume (mm^3^), changes in hemoglobin, hematocrit, plasma sodium, blood pressure, and post-biopsy eGFR	Pre-biopsy intranasal desmopressin administration in patients with impaired renal function markedly decreased the occurrence and size of perirenal hematomas, confirming its safety and efficacy
Manno (2011)	Double-blind randomized controlled clinical trial	Italy	August 2008–December 2009	Age: 16–80 years Serum creatinine ≤ 1.5 mg/dL eGFR ≥ 60 mL/min/1.73 m^2^ Normal coagulation parameters	80	(Subcutaneously, dosage of 0.3 μg/kg	1 h before the kidney biopsy	82	Saline	24 h & in hematoma 48 & 72 h	Incidence of post-biopsy bleeding (major: AV fistula, obstruction, transfusion, embolization, sepsis, or death; minor: hematoma ≥ 20 × 20 mm, gross hematuria)	Hematoma size, post-biopsy hemoglobin, coagulation profile, GFR, blood pressure, hospital sta	Administering desmopressin before percutaneous kidney biopsy reduces bleeding risk and hematoma size without elevating overall costs

**Table 2:** Baseline characteristics of the included studies.

Study ID	Arm	Ppts (%)	Age (y)	Gender (% male)	BMI (kg/m^2^)	eGFR (mL/min/1.73 m^2^)	Coagulation profile					Cr (mg/dL)	S. Na (mEq/L)	Blood pressure	
							Hb (g/dL)	Plt (× 10^3^/µL)	PT (ratio)	aPTT (s)	Bleeding Time (min)			Systolic	Diastolic
Chakrabarti (2025)	Desmopressin group	74 (48.7%)	39 ± 15	34 (46%)	21.8 ± 3.7	23 ± 13	9.1 ± 1.8	265 ± 120	1.08 ± 0.12	29 ± 6	0.73 ± 0.1	3.6 ± 1.5	132 ± 4	131 ± 10	82 ± 9
	Placebo group	78 (51.3%)	39 ± 16	44 (56%)	22.2 ± 3.7	22 ± 12	9.4 ± 2.0	244 ± 104	1.07 ± 0.09	30 ± 6	0.77 ± 0.2	3.7 ± 1.5	133 ± 4	132 ± 9	83 ± 8
Prasad (2025)	Desmopressin group	101 (49.8%)	38.53 ± 15.10	67 (66.33%)	24.87 ± 7.63	40.17 ± 41.74	10.73 ± 2.04	201.54 ± 9.71	*	28.15 ± 3.60	–	2.47 ± 1.96	133.48 ± 6.76	118.76 ± 8.05	76.44 ± 5.45
	Placebo group	102 (50.2%)	42.64 ± 15.13	60 (58.82%)	24.44 ± 5.19	37.40 ± 38.95	10.30 ± 1.76	207.45 ± 11.22	reported in seconds	28.69 ± 3.32	–	2.66 ± 2.33	131.68 ± 6.85	118.00 ± 8.60	77.89 ± 5.89
Sethi (2024)	Desmopressin group	40 (50%)	42.90 ± 14.35	26 (65%)	–	22.41 ± 12.37	10.69 ± 2.04	2.47 ± 0.97	*	32.28 ± 11.39	2.62 ± 0.39	4.19 ± 4.36	–	–	–
	Placebo group	40 (50%)	45.25 ± 11.00	24 (60%)	–	19.23 ± 12.86	9.55 ± 1.40	2.47 ± 1.13	*	30.23 ± 7.44	2.53 ± 0.50	4.65 ± 2.83	–	–	–
Sattari (2022)	Desmopressin group	60 (50%)	43.73 ± 16.88	31 (51.7%)	25.98 ± 2.73	54.14 ± 20.52	11.39 ± 2.02	289 ± 89	1.01 ± 0.05	–	–	1.57 ± 0.81	140.83 ± 2.68	125.83 ± 11.5	78.33 ± 6.15
	Placebo group	60 (50%)	46.85 ± 14.94	27 (45%)	25.45 ± 3.38	49.41 ± 15.63	11.02 ± 1.9	275 ± 70	1.04 ± 0.07	–	–	1.92 ± 1.2	140 ± 3.59	125.74 ± 12.14	79.5 ± 7.46
Manno (2011)	Desmopressin group	80 (49.4%)	39.5 ± 14.2	45 (56.3%)	–	94.2 ± 22.8	13.9 ± 1.8	261.67 ± 56.62	1.0 ± 0.1	–	4.6 ± 1.2	1.0 ± 0.3	–	126.8 ± 13.6	80.9 ± 9.3
	Placebo group	82 (50.6%)	41.7 ± 15.0	43 (52.4%)	–	89.4 ± 21.3	13.5 ± 1.8	250.67 ± 60.37	1.0 ± 0.1	–	4.5 ± 1.2	1.0 ± 0.2	–	128.3 ± 12.5	82.6 ± 9.3

Values are mean ± SD unless specified(-) indicate data not reported,(*) indicate reported in secondsAbbreviations: Ppts = Participants, BMI Body mass index, eGFR = Estimated glomerular filtration rate,Hb = Hemoglobin, Plt = Platelet count, Cr = Creatinine, S. Na = Serum sodium.

**Table 3:** A summary table with key meta-analytic outcomes.

Outcome	No. of studies	Participants	Effect estimate	95% CI	*p* value	I^2^ (%)
Total bleeding events	3	157	RR = 0.51	[0.31, 0.83]	0.008	68
Gross hematuria	4	555	RR = 1.03	[0.40, 2.63]	0.95	53
Hematoma formation	5	717	RR = 0.60	[0.30, 1.19]	0.15	78
Need for blood transfusion	3	435	RR = 1.57	[0.67, 3.67]	0.30	0
Need for intervention (radiological/surgical)	3	435	RR = 1.81	[0.38, 8.68]	0.46	0
Flushing incidence	2	323	RR = 2.09	[0.27, 16.02]	0.48	0
Systolic blood pressure	3	434	MD = 0.97	[−1.41, 3.36]	0.42	0
Diastolic blood pressure	3	434	MD = 0.86	[−0.67, 2.39]	0.27	0
Hemoglobin level	3	434	MD = -0.06	[−0.21, 0.09]	0.44	78

RR: Risk Ratio , MD: Mean Difference,  \(I^{2}\) : heterogenity index

### Assessment of the risk of bias

The included RCTs were evaluated using ROB 2. The assessment indicated that four studies demonstrated low risk of bias across all domains, while Sattari 2022 showed high risk in the third domain. A detailed illustration of the risk of bias for the included studies is presented in Fig. S1. Assessment of publication bias using funnel plots or Egger’s regression was not undertaken. Regarding statistical guidance from key sources (including the JBI Manual and Cochrane Handbook), current recommendations advise against these methods when fewer than 10 studies are available due to the unreliability and low power of such assessments. Therefore, the possibility of publication bias cannot be excluded.

### Efficacy of desmopressin

**Fig. 2:**
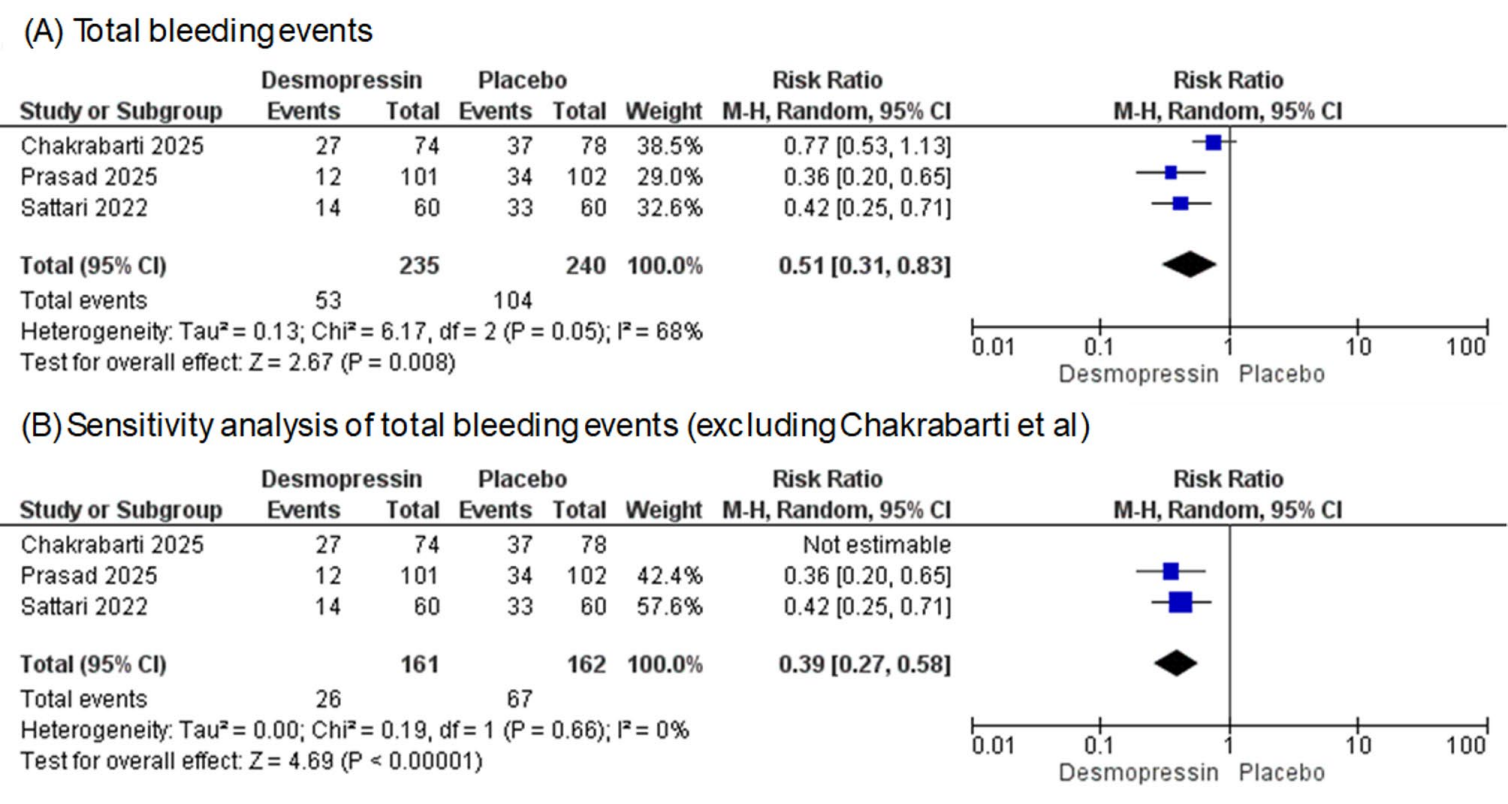
Forest plots of efficacy outcomes: (**A**) Total bleeding events. (**B**) Sensitivity analysis of total bleeding events (excluding Chakrabarti et al.).

Three studies evaluated total bleeding events with a total of 157 participants. The pooled effect statistically favors desmopressin over placebo (RR = 0.51, 95% CI [0.31, 0.83], *P* = 0.008) (Fig. 2). There was substantial heterogeneity (I^2^ = 68%). We conducted a sensitivity analysis to explore the source of heterogeneity. The heterogeneity was resolved by excluding Chakrabarti et al. (RR = 0.39, 95% CI [0.27, 0.58], *P* < 0.00001, I^2^ = 0%) (Fig. 1). Regarding gross hematuria, our analysis included four studies with 555 participants. The combined RR demonstrates no statistically significant difference between the two groups; the total RR = 1.03 (95% CI [0.4–2.63], *p* = 0.95) (Fig. S2). Concerning hematoma formation, our analysis encompassed five studies. The combined RR was 0.6 (95% CI [0.3, 1.19], *p* = 0.66), indicating no statistically significant improvement in either group (Fig. S2). The assessment of heterogeneity yielded I^2^ = 78%, suggesting significant heterogeneity. Sensitivity analysis excluding Sethi et al. resolved the heterogeneity, and the pooled effect estimate favors desmopressin (RR 0.42, 95% CI [0.31, 0.58], *P* < 0.00001, I^2^ = 0%) (Fig. S2). Two studies assessed Hematoma size; the pooled analysis revealed no significant difference between the two groups (MD = −3.39, 95% CI [−7.35, 0.57], *P* = 0.09, I^2^ = 94%) (Fig. S2). About the need for blood transfusion, our analysis encompassed three studies. The combined RR was 1.57 (95% CI [0.67, 3.67], *p* = 0.30, I^2^ = 0%) (Fig. S2), indicating no statistically significant difference between the groups. Concerning the need for an interventional or radiological procedure, our analysis included three studies. As shown in Fig. S2, overall RR was 1.81 (95% CI [0.38, 8.68], *p* = 0.46, I^2^ = 0%), meaning that there was no statistically significant difference between the two groups. Certainty of the evidence by GRADE ranged from high to Low across outcomes. For total bleeding events and hematoma formation, the certainty of evidence was high as the methodologies, follow up and assessment tools were highly similar across studies with accurate measurements. Need for Blood transfusion and Interventional radiology was rated as moderate due to contradictory findings of included studies. Gross hematuria was rated as low because of the wide confidence interval and high level of imprecision (Fig. S4).

### Safety outcomes

Two studies demonstrate that there was no statistically significant difference between the two groups in the incidence of flushing (RR = 2.09, 95% CI [0.27–16.02], *p* = 0.48) (Fig. S3). The pooled analysis of three studies demonstrated comparable rates of any systolic and diastolic blood pressure between both groups (MD = 0.93, 95% CI [−1.41, 3.38], *P* = 0.42, I^2^ = 0%) (Fig. S3), (MD = 0.86, 95% CI [−0.67, 2.39], *P* = 0.27, I^2^ = 0%), respectively (Fig. S3). Three studies assessed hemoglobin level; the pooled analysis revealed no significant difference between the two groups (MD = −0.06, 95% CI [−0.21, 0.09], *P* = 0.44, I^2^ = 78%) (Fig. S3). Sensitivity analysis by excluding Manno et al. resolved the heterogeneity, and the pooled effect estimate favors the placebo (MD = −0.39, 95% CI [−0.65, −0.12], *P* = 0.004, I^2^ = 0%) (Fig. S3).

## Discussion

This meta-analysis and systematic review evaluated the safety and efficacy of desmopressin in patients undergoing native renal biopsy, based on evidence from five randomized controlled trials with diverse patient groups and study designs. There was a lack of trials that had enrolled adults with controlled blood pressure and excluded patients with significant comorbidities or coagulation disorders. The trials used were, on the whole, well-matched at baseline with similar demographic and clinical characteristics in control and intervention groups. There was heterogeneity in dosing (0.3–3 mcg/kg), route of administration (subcutaneous, intranasal), and timing (all within 1 h before biopsy), but no suggestion of a perceivable pattern between regimen and effectiveness. There was heterogeneity in the definition and measurement of some outcomes, like hematoma size and gross hematuria, possibly resulting in heterogeneity in pooled analyses.

Three trials with a total of 157 patients examined the total bleeding events^18,19,21^. Pooled analysis was in favor of desmopressin over placebo, but highly significant heterogeneity existed (I^2^ = 68% % %). Excluding the trial^18^, which exclusively involved patients with normal renal function (eGFR ≥ 60 mL/min/1.73 m^2^), resolved the heterogeneity. This suggests that the phenotype of study populations, most significantly renal function, may influence the efficacy of desmopressin. Notably, Chakrabarti et al. showed no clinical effect in those with intact renal function, as would be expected in their conclusion that desmopressin did not reduce bleeding in this subgroup^18^. According to a retrospective analysis, patients with serum creatinine < 1.8 mg/dL had higher risks of bleeding due to post-biopsy decrease in hemoglobin, while desmopressin did not reduce bleeding^22^. Furthermore, a retrospective analysis of patients with eGFR < 30 mL/min/1.73 m^2^ having percutaneous kidney biopsy under ultrasound guidance revealed that prebiopsy intranasal desmopressin administration was linked to a considerably decreased overall incidence of bleeding problems^23^.

Four trials that included 555 participants reported gross hematuria^18–21^. The pooled risk ratio indicated no difference between desmopressin and placebo. The data analysis indicated that all trials had low event rates and that results were consistent, irrespective of route or dose of desmopressin. For example, Manno et al. and Prasad et al. using various delivery routes (subcutaneous and intranasal, respectively), showed similar percentages of gross hematuria in the two groups. The formation of a hematoma was assessed in five studies. The overall RR was 0.6 with significant heterogeneity (I^2^ = 78% % %). Sensitivity analysis excluding Sethi et al. result (which had an extremely high event rate in the desmopressin arm) left a strongly significant effect for desmopressin, illustrating how heterogeneity in outcome definitions (e.g., hematoma size cut-offs) can conceal genuine effects^7^. Sethi et al. found that only patients with eGFR ≤ 45 mL/min/1.73 m^2^ were selected, resulting in higher hematomas in the desmopressin arm. This contradicts previous studies and may be related to a limited sample size or patient selection^7^. Notably, Rao et al. reported a 50% reduction in perinephric hematomas with intranasal desmopressin in renal impairment patients, suggesting high-risk subgroup benefits^24^.

Two studies compared hematoma size across groups and found no significant difference^19,21^. Both trials measured hematoma size at 24 h post-injury, although differences in measuring units (mm^2^ vs. cm^3^) and reporting time may have contributed to the significant heterogeneity. Three studies additionally examined the transfusion need and intervention technique. Data analysis revealed no substantial distinction between transfusions and interventional/radiological treatments. Data extraction revealed that the occurrences were infrequent in all investigations, reducing the ability to detect differences. This concurs with a large retrospective cohort study by Leclerc et al. who found the same incidence of symptomatic hematomas and intervention in patients receiving desmopressin as those who did not receive desmopressin^25^.

The evidence does not favor prophylactic desmopressin administration in all patients undergoing native renal biopsy. The benefit appears restricted to reductions in minor bleeding episodes, which may not justify the use of associated costs and monitoring requirements. However, certain populations—e.g., those with compromised renal function (eGFR < 60 mL/min/1.73 m^2^) or at elevated risk for bleeding—may derive greater benefit^24^. Radhakrishnan et al., on the other hand, detected no statistically significant decrease in bleeding events with desmopressin in any eGFR subgroup, although there is a suggestion of a potential but unproven benefit in the most vulnerable patients in the lowest eGFR group (0/5 vs. 3/10)^26^. For example, Manno et al. demonstrated a 60% reduction in hematoma formation in patients with serum creatinine > 132.4 μmol/L, while this subgroup analysis is to be confirmed in further trials^19^. Eventually, the efficacy of desmopressin is situation-dependent. Regarding Chakrabarti et al. and Manno et al. there is little utility in low-risk, normal renal function patients^18,19^. Conversely, Sattari et al., Prasad et al. reported clear benefit in moderate CKD or mixed populations^20,21^. On the other hand, Sethi et al. addressed possible harm in severe CKD. This highlights the role of patient selection for desmopressin prophylaxis in renal biopsy. The data extraction forms highlight the need for risk assessment on an individual basis rather than routine use.

Pooled analyses of available safety outcomes, including flushing, blood pressure, and hemoglobin changes, did not demonstrate statistically significant differences between desmopressin and placebo, suggesting a comparable short-term safety profile based on the limited data reported across randomized controlled trials. Specifically, the flushing incidence was no different between groups, and comparison of pooled systolic and diastolic blood pressure indicated negligible mean differences (0.93 mmHg and 0.86 mmHg, respectively), suggesting a neutral hemodynamic profile. Regarding fluid-related safety, desmopressin-induced hyponatremia is a recognized adverse event in other clinical contexts. However, our review identified no studies reporting sodium levels in patients receiving desmopressin before renal biopsy, highlighting a critical gap in data. To address this, the included studies employed changes in hemoglobin concentration as a potential indirect indicator to assess potential water retention or hemodilution, given desmopressin’s known effects on fluid balance. Across these studies, no significant hemoglobin changes were detected, aside from sensitivity analysis, excluding one trial, which showed a modest reduction in favor of the placebo, suggesting that the doses used may not induce clinically relevant fluid shifts or dilutional effects. This lack of direct sodium monitoring, however, remains a significant limitation, potentially underestimating the full safety profile of desmopressin in this setting^26,27^.

Besides, the absence of excess thrombotic or severe cardiovascular events within this review also fits with the general literature, which as a whole regards desmopressin to be hemodynamically safe, even though rare thrombotic side effects have been found in high-risk patients^28–30^. In summary, while the reassuring safety findings in this review are commensurate with controlled trial outcomes, they may not accurately reflect the true risk of hyponatremia and rare side effects associated with broader clinical use, emphasizing the importance of patient selection and monitoring following administration when desmopressin is used prophylactically for renal biopsy. We also acknowledge the extensive efforts of the KHA-CARI Guideline recommendations for renal biopsy and evidence-based clinical guidelines for desmopressin, as published until 2017^31^. The KHA-CARI guidelines analyzed randomized and observational studies extensively. They concluded that while desmopressin could reduce perinephric hematomas by imaging, there was no substantial evidence to employ it prophylactically in routine for the prevention of clinically important bleeding. They also emphasized the need for further well-powered studies due to sample size constraints, heterogeneity of results, and scant safety information. Our systematic review and meta-analysis contribute to and build upon this foundation by including late-published randomized controlled trials after the KHA-CARI evidence cutoff date, applying stringent inclusion criteria for native kidney biopsies, and conducting a formal GRADE analysis of the certainty of evidence. While our findings generally support the conservative stance of the KHA-CARI guideline on the limited benefit in clinically important bleeding outcomes and unresolved safety issues, the updated analysis provides a quantitative synthesis of evidence and a rigorous assessment of study quality and heterogeneity. Together, these two complementary articles emphasize the continuing need for larger, well-designed randomized trials with standardized bleeding definitions and vigilant safety monitoring to establish the place of desmopressin in this setting. The existence of heterogeneity in dose and route of desmopressin administration among included trials is acknowledged as a source of clinical variability. Sensitivity analyses suggest this did not directly confound efficacy results to a greater extent than underlying patient risk factors, such as renal function. However, future randomized controlled trials should address the question of optimal dose and route through harmonized, sufficiently powered, and prospectively stratified studies.

### Safety consideration

One of the significant limitations of the current evidence is the lack of systematic recording of post-DDAVP serum sodium values. This is particularly warranted because desmopressin, a synthetic analogue of vasopressin, is known to cause water retention with consequent hyponatremia in the most vulnerable populations, such as those with chronic kidney disease or acute kidney injury. Although some studies reviewed, as shown in Table 1, monitored baseline or isolated post-administration sodium levels, these measurements were made neither routinely nor with adequate power to detect meaningful differences between groups. As sodium levels are not being monitored appropriately, it is impossible to reach firm conclusions about this potentially clinically significant adverse effect, and it may also underestimate the incidence and severity of hyponatremia in clinical practice. Further research should include strict monitoring of serum sodium levels to determine the risk–benefit ratio of desmopressin in this situation accurately.

### Clinical implications

This meta-analysis suggests that desmopressin reduces the incidence of total bleeding events after native renal biopsy compared with placebo, but is ineffective in reducing clinically significant complications, such as hematoma or the need for transfusion. Hence, the routine administration of desmopressin is not recommended in all native renal biopsy patients. But in the absence of evidence of a safety problem, clinicians could consider desmopressin as an individualized therapy for those at particularly high risk for bleeding. Decisions should weigh the limited potential benefit against considerations such as cost and the need for careful patient selection, pending further research in specific populations. The theoretical risk of hyponatremia associated with desmopressin administration warrants careful follow-up of serum sodium both in clinical practice and in future research protocols. This monitoring, especially in the first hours following administration, would clarify the safety profile of desmopressin, including in patients with baseline renal disease or other risk factors for electrolyte imbalances.

### Study limitations and methodological considerations

There are several limitations to interpreting our results. First, the considerable heterogeneity in main outcomes required sensitivity analyses to be performed, omitting individual studies, which may have limited the generalizability of our findings. Only a couple of the included RCTs provided formal sample size calculations or were specifically powered to detect clinically meaningful differences in primary bleeding outcomes. The total number of included patients (717 across five RCTs) means that, while some efficacy signals were observed (such as a reduction in total bleeding events), the overall precision of effect estimates was limited—especially for major bleeding and safety endpoints, where confidence intervals were wide and statistical significance was not consistently reached. These limitations underscore the need for adequately powered, multicenter studies with standardized outcome definitions to determine whether desmopressin offers a true therapeutic advantage in this setting. The variation in definitions of bleeding complications across the studies, ranging from gross hematuria to clinically relevant bleeding requiring intervention, also complicated the interpretation of pooled effect estimates. The comparatively brief follow-up durations (only 24 h) in the majority of the included studies would have overlooked delayed adverse events or bleeding complications. Furthermore, the lack of standardization between studies regarding post-biopsy outcomes, other outcomes, and interventions created bias in measuring outcomes. The limited sample sizes in individual studies, especially, may have contributed to the failure to detect statistically significant differences in main safety outcomes, such as severe hyponatremia. The Prasad et al. (2025) trial, although the largest in our review with 203 patients, still demonstrated limited power to detect rare but clinically significant adverse events. One of the significant limitations of the available evidence is the absence of a systematic presentation of post-DDAVP serum sodium measurements, which renders it infeasible to conduct a robust assessment of a possible severe adverse effect. Absence of such data is a crucial flaw in the safety assessment and needs to be addressed in future research. One significant limitation of our analysis is that all the trials included in our study had evaluated desmopressin use before native kidney biopsy. Therefore, our findings and recommendations cannot be extended to kidney transplant patients; future research will need to establish the efficacy and safety of desmopressin in transplant kidney biopsies. Inadequate standardized definitions for endpoints, such as gross hematuria and hematoma size, in the included studies posed a major challenge to our meta-analysis, leading to significant heterogeneity (I^2^ = 78% for hematoma formation, I^2^ = 94% for hematoma size). For example, Manno et al. (2011) measured hematoma size in mm^2^, while Sattari et al. (2022) used cm^3^; neither study provided clear definitions for gross hematuria or employed a standardized scale, such as CTCAE v5.0, to measure outcome severity. Such heterogeneity will have had an impact on the pooled effect estimates. To mitigate this, we established operational definitions (e.g., gross hematuria, as clinically documented and observable hematuria) and conducted sensitivity analyses to control for heterogeneity sources, as outlined in the Results. Standardized frameworks, such as CTCAE v5.0, should be utilized in future studies to classify bleeding outcomes and establish uniform measurement protocols, thereby enhancing comparability. However, considering our institution’s current resource limitations in Egypt, we are unable to access Embase directly, nor can we hire the services of a skilled, dedicated medical librarian to conduct systematic review searches. Nevertheless, with these limitations in mind, we have made our best efforts to conduct a thorough and systematic search using the resources available to us, including PubMed (utilizing MeSH terms), Web of Science, Scopus, the Cochrane Library, and ClinicalTrials.gov. Due to the limited number of studies available and the differences in patient populations and reporting in these studies, a formal meta-regression or stratified analysis by dose or route of administration could not be performed. This limits the accuracy of our estimates of the comparative effectiveness or safety of different dosing strategies.

### Future research directions

Our analysis has highlighted multiple priority areas for research. We need to identify large-scale randomized controlled clinical trials with sufficient power to detect both efficacy and safety outcomes, particularly concerning patients with varying degrees of kidney dysfunction. Future randomized controlled trials should prospectively monitor serum sodium levels following desmopressin administration. A pre- and post-administration electrolyte measurement protocol would enable a more accurate determination of the risk of hyponatremia and inform clinical practice regarding the use of desmopressin for native renal biopsy. Follow-up research must establish whether the harms and benefits of desmopressin vary among patient subgroups, such as those with advanced chronic kidney disease or those on anticoagulants. This may be achieved through subgroup analysis in larger trials or by conducting independent trials in these subgroups. This would determine if individualizing the use of desmopressin to a patient’s risk profile for bleeding and harm may maximize benefit and minimize harm. Finally, research to address comparative-effectiveness approaches to desmopressin dosing (e.g., dosing regimens, routes of dosing (e.g., subcutaneous vs. IV), and timing (e.g., pre-procedure) would assist in optimizing therapies in clinical protocol. The construction and validation of bleeding risk prediction models may delineate patients most likely to benefit from prophylactic desmopressin while minimizing unnecessary exposure in lower-risk individuals. These models could also allow recognition of relevant baseline kidney function, coagulation parameters, and clinical variables (even if described are evident of an increased bleeding risk. Given the safety issues highlighted, we also recommend future studies on other prophylactic strategies, such as a modification to the biopsy technique, alternative hemostatic agents, and strategies of risk stratification to put desmopressin only in higher-risk patients. The addition of point-of-care coagulation testing, facilitating prophylactic interventions, is another strategy worth exploring.

## Conclusion

In this study, desmopressin administration in patients undergoing native renal biopsy was associated with a significant reduction in total bleeding risk. However, this benefit did not extend to specific clinically significant outcomes such as hematoma formation, major bleeding, need for nephrectomy, or blood transfusion. Notably, the observed reduction in total bleeding risk was extrapolated from only three trials comprising a total of 157 patients, which limits the generalizability and robustness of this finding. Without further large randomized controlled trials confirming a meaningful clinical benefit, desmopressin cannot be recommended for routine use. Therefore, its application should be carefully considered, especially in the context of risk–benefit balance and cost-effectiveness.

## Supplementary Information

Below is the link to the electronic supplementary material.

Supplementary Material 1

## Data Availability

All data generated or analyzed during this study are included in this published article and its supplementary information files.
