# Supplementary material for: Effect of desmopressin on bleeding outcomes after native renal biopsy: a systematic review and meta-analysis
Source: Sci Rep. 2025 Nov 7;15:39005. doi: 10.1038/s41598-025-24092-7 (PMC12594780; doi:10.1038/s41598-025-24092-7)
Supplement: Supplementary file 1 — Supplementary Material 1 [file 41598_2025_24092_MOESM1_ESM.docx]

Effect of Desmopressin on Bleeding Outcomes after Native Renal Biopsy – A Systematic Review and Meta-analysis

Ammar Yasser Ali^1^, Rem Ehab Abdelkader^1^, Rashad G. Mohamed^1^, Mohammed. N. Abdelaziz^2^, Radwa M. Abdelsattar^1^, Ahmed R. A. Moustafa^1^, Mohamed Rizk Elsayed^1^, Bassant Barakat^2^, Emad Samaan^3^

1. Mansoura Manchester Medical Program for Medical Education, Faculty of Medicine, Mansoura University, Mansoura, Egypt
2. Faculty of Medicine, Mansoura University Hospital, Mansoura, Egypt.
3. Associate Professor of Nephrology, Mansoura Faculty of Medicine- Egypt.

*Correspondence: Mohammed. N. Abdelaziz: Medical Intern, Faculty of Medicine, Mansoura University, Egypt. Postal address: Arab Republic of Egypt- Al-Daqahlia Governorate- Mansoura- Mansoura. Postal office number 35516. E-mail: mohammednasser@std.mans.edu.eg. ORCID: - 0000-0003-2699-2901.

Supplementary Materials - Index

| Supplementary Appendixes | |
| --- | --- |
| 1. Search strategy | Page 2 |
| Supplementary Figures | |
| 1. Quality assessment of RCT using ROB2 too | Page 5 |
| 1. Forest plots of efficacy outcomes | Page 6 |
| 1. Forest plots of safety outcomes | Page 7 |
| 1. GRADE summary of findings for the impact of intranasal desmopressin compared to placebo in reducing post-biopsy bleeding complications. | Page 8 |

1. Search strategy

All database searches (PubMed, Cochrane Central Register of Controlled Trials, Scopus, Web of Science, and ClinicalTrials.gov) were completed on May 7th, 2025.

1. Database: PubMed 1979 to Present

Search Strategy:

| Search | Query | Results |
| --- | --- | --- |
| #7 | Search: #3 AND #6 Sort by: Publication Date | [232](https://pubmed.ncbi.nlm.nih.gov/?term=%233+AND+%236&sort=pubdate&ac=no) |
| #6 | Search: #4 AND #5 Sort by: Publication Date | [910,467](https://pubmed.ncbi.nlm.nih.gov/?term=%234+AND+%235&sort=pubdate&ac=no) |
| #5 | Search: "kidney"OR"renal"OR"Tissue"OR"percutaneous" Sort by: Publication Date | [3,654,584](https://pubmed.ncbi.nlm.nih.gov/?term=%22kidney%22OR%22renal%22OR%22Tissue%22OR%22percutaneous%22&sort=pubdate&ac=no) |
| #4 | Search: Biopsy Sort by: Publication Date | [4,610,873](https://pubmed.ncbi.nlm.nih.gov/?term=Biopsy&sort=pubdate&ac=no) |
| #3 | Search: #1 OR #2 Sort by: Publication Date | [7,012](https://pubmed.ncbi.nlm.nih.gov/?term=%231+OR+%232&sort=pubdate&ac=no) |
| #2 | Search: (1-deamino-8-D-arginine vasopressin or 1-desamino-8-arginine vasopressin or adiuretin or adiuretin SD or apo-desmopressin or DDAVP or desmogalen or desmopressin or desmopressin acetate or desmopressin monoacetate or desmopressin monoacetate, trihydrate or desmopressine ferring or desmospray or desmotabs or minirin or minurin or nocutil or octim or octostim or vasopressin, deamino arginine) Sort by: Publication Date | [7,011](https://pubmed.ncbi.nlm.nih.gov/?term=%281-deamino-8-D-arginine+vasopressin+or+1-desamino-8-arginine+vasopressin+or+adiuretin+or+adiuretin+SD+or++apo-desmopressin+or+DDAVP+or+desmogalen+or+desmopressin+or+desmopressin+acetate+or+desmopressin+monoacetate+or+desmopressin+monoacetate%2C+trihydrate+or+desmopressine+ferring+or+desmospray+or+desmotabs+or+minirin+or++minurin+or+nocutil+or+octim+or+octostim+or+vasopressin%2C+deamino+arginine%29&sort=pubdate&ac=no) |
| #1 | Search: deamino arginine vasopressin Sort by: Publication Date | [4,812](https://pubmed.ncbi.nlm.nih.gov/?term=deamino+arginine+vasopressin&sort=pubdate&ac=no) |

Total 232 Studies

2. Database: Cochrane Central Register of Controlled Trials

| #1 | Deamino Arginine Vasopressin | 523 |
| --- | --- | --- |
| #2 | "1-deamino-8-D-arginine vasopressin"OR"1-desamino-8-arginine vasopressin"OR"adiuretin"OR"adiuretin SD"OR"apo-desmopressin"OR"DDAVP"OR"desmogalen"OR"desmopressin"OR"desmopressin acetate"OR"desmopressin monoacetate "OR"ddesmopressin monoacetate, trihydrate"OR"desmopressine ferring "OR"desmospray"OR"desmotabs"OR"minirin"OR"minurin"OR"nocutil"OR"octim"OR"octostim"OR"vasopressin, deamino arginine" | 1061 |
| #3 | #1 OR #2 | 1085 |
| #4 | Biopsy | 34846 |
| #5 | "kidney"OR"renal"OR"Tissue"OR"percutaneous" | 264300 |
| #6 | #4 AND #5 | 15704 |
| #7 | #6 AND #3 | 34 |

Search Strategy:

Total: 34 Studies

3. Database: Scopus

Search strategy:

("Deamino Arginine Vasopressin"OR"1-deamino-8-D-arginine vasopressin"OR"1-desamino-8-arginine vasopressin"OR"adiuretin"OR"adiuretin SD"OR"apo-desmopressin"OR"DDAVP"OR"desmogalen"OR"desmopressin"OR"desmopressin acetate"OR"desmopressin monoacetate "OR"ddesmopressin monoacetate, trihydrate"OR"desmopressine ferring "OR"desmospray"OR"desmotabs"OR"minirin"OR"minurin"OR"nocutil"OR"octim"OR"octostim"OR"vasopressin, deamino arginine")AND(Biopsy)AND("kidney"OR"renal"OR"Tissue"OR"percutaneous")

Results: 536

4. Database: Web Of Science

Search Strategy:

| #1 | ALL=(deamino arginine vasopressin ) | 891 |
| --- | --- | --- |
| #2 | (ALL=(deamino arginine vasopressin )) OR ALL=((1-deamino-8-D-arginine vasopressin or 1-desamino-8-arginine vasopressin or adiuretin or adiuretin SD or apo-desmopressin or DDAVP or desmogalen or desmopressin or desmopressin acetate or desmopressin monoacetate or desmopressin monoacetate, trihydrate or desmopressine ferring or desmospray or desmotabs or minirin or minurin or nocutil or octim or octostim or vasopressin, deamino arginine) ) | 7317 |
| #3 | #1 OR #2 | 7317 |
| #4 | (#1 OR #2) AND ALL=(Biopsy) | 141 |
| #5 | ((#1 OR #2) AND ALL=(Biopsy)) AND ALL=("kidney"OR"renal"OR"Tissue"OR"percutaneous" ) | 71 |

Total = 71

5. Database: clinical trial. gov

("Deamino Arginine Vasopressin"OR"1-deamino-8-D-arginine vasopressin"OR"1-desamino-8-arginine vasopressin"OR"adiuretin"OR"adiuretin SD"OR"apo-desmopressin"OR"DDAVP"OR"desmogalen"OR"desmopressin"OR"desmopressin acetate"OR"desmopressin monoacetate "OR"ddesmopressin monoacetate, trihydrate"OR"desmopressine ferring "OR"desmospray"OR"desmotabs"OR"minirin"OR"minurin"OR"nocutil"OR"octim"OR"octostim"OR"vasopressin, deamino arginine") AND (Biopsy) AND ("kidney"OR"renal"OR"Tissue"OR"percutaneous")

Result: 4 Studies

* We recommend the PRESS 2015 checklist as a future tool to ensure rigorous, peer-reviewed search strategy development.


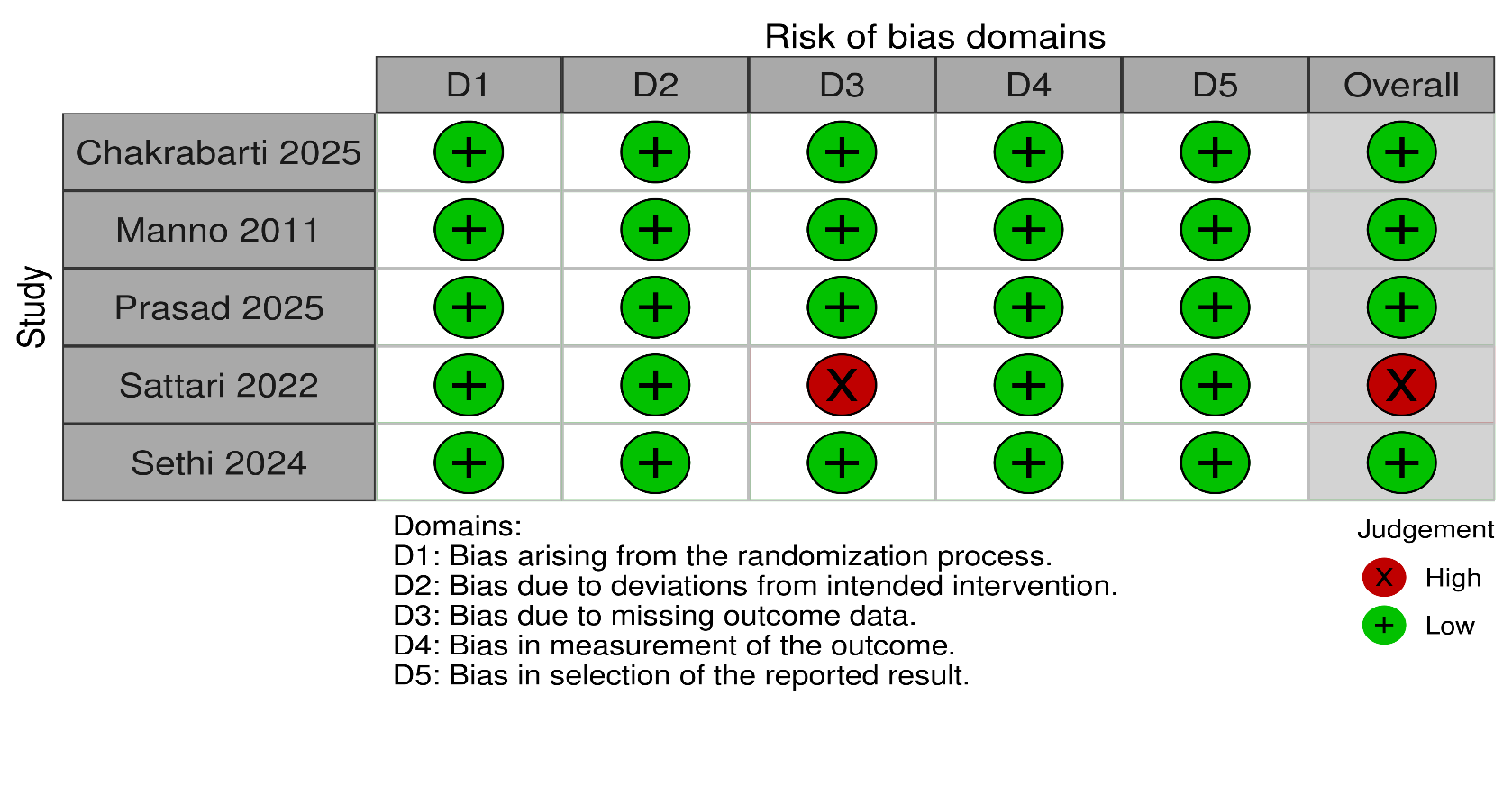


Figure S1: Quality assessment of RCT using ROB2 too

**Figure S2: Forest plots of efficacy outcomes (A) gross hematuria. (B) Hematoma formation. (C) Sensitivity analysis of hematoma formation (excluding Sethi et al). (D) The need for blood transfusion. (E) The need for an interventional or radiological procedure**

Figure S3: Forest plots of safety outcomes: (A) Flushing. (B) Change in systolic blood pressure. (C) Change in diastolic blood pressure. (D) Change in hemoglobin level. (E) Sensitivity analysis of changes in hemoglobin level (excluding Manno et al)


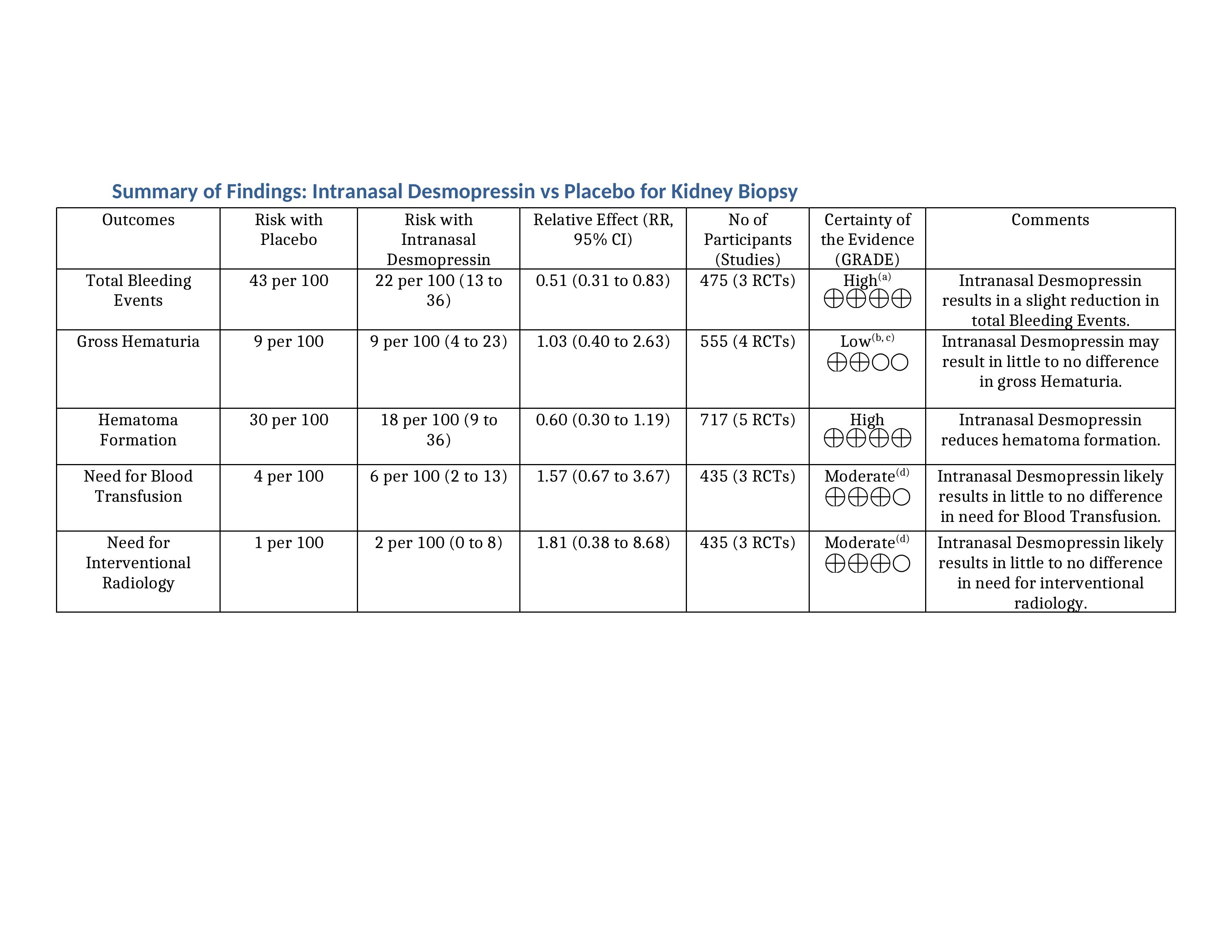


Figure S4: GRADE summary of findings for the impact of intranasal desmopressin compared to placebo in reducing post-biopsy bleeding complications.

## Explanatory Notes

- a. Only one study (Sattari 2022) had some missing data without explanation.
- b. Two studies were in favor of the intervention while the other two were against.
- c. The confidence interval spans all possible outcomes (slight benefit, no benefit, and harm), indicating a high level of imprecision likely due to contradictory findings of the included studies.
- d. Due to contradictory findings of included studies.

## GRADE Working Group Definitions

- High certainty: We are very confident that the true effect lies close to that of the estimate of the effect.
- Moderate certainty: We are moderately confident in the effect estimate: the true effect is likely to be close to the estimate, but there is a possibility that it is substantially different.
- Low certainty: Our confidence in the effect estimate is limited: the true effect may be substantially different from the estimate of the effect.
- Very low certainty: We have very little confidence in the effect estimate: the true effect is likely to be substantially different from the estimate of the effect.
